# Characterization of the Biofilms Formed by Histamine-Producing *Lentilactobacillus parabuchneri* Strains in the Dairy Environment

**DOI:** 10.3390/foods12071503

**Published:** 2023-04-03

**Authors:** Agustina Sarquis, Diellza Bajrami, Boris Mizaikoff, Victor Ladero, Miguel A. Alvarez, Maria Fernandez

**Affiliations:** 1 Departmento de Tecnología y Biotecnología de Productos Lácteos, Instituto de Productos Lácteos de Asturias, IPLA, CSIC, 33300 Villaviciosa, Spain; 2 Instituto de Investigación Sanitaria del Principado de Asturias (ISPA), 33011 Oviedo, Spain; 3 Institute of Analytical and Bioanalytical Chemistry, Ulm University, Albert Einstein-Allee 11, 89081 Ulm, Germany; 4 Hahn-Schickard, Institute for Microanalysis Systems, Sedanstrasse 14, 89077 Ulm, Germany

**Keywords:** biogenic amines, histamine, *Lentilactobacillus parabuchneri*, biofilms

## Abstract

*Lentilactobacillus parabuchneri*, a lactic acid bacterium, is largely responsible for the production and accumulation of histamine, a toxic biogenic amine, in cheese. *L. parabuchneri* strains can form biofilms on the surface of industry equipment. Since they are resistant to cleaning and disinfection, they may act as reservoirs of histamine-producing contaminants in cheese. The aim of this study was to investigate the biofilm-producing capacity of *L. parabuchneri* strains. Using the crystal violet technique, the strains were first categorized as weak, moderate or strong biofilm producers. Analysis of their biofilm matrices revealed them to be mainly composed of proteins. Two strains of each category were then selected to analyze the influence on the biofilm-forming capacity of temperature, pH, carbon source, NaCl concentration and surface material (i.e., focusing on those used in the dairy industry). In general, low temperature (8 °C), high NaCl concentrations (2–3% *w*/*v*) and neutral pH (pH 6) prevented biofilm formation. All strains were found to adhere easily to beech wood. These findings increase knowledge of the biofilm-forming capacity of histamine-producing *L. parabuchneri* strains and how their formation may be prevented for improving food safety.

## 1. Introduction

A biofilm is a community of bacterial cells enclosed in a self-produced polymeric matrix adhered to an inert or living surface [1,2,3]. The composition of the biofilm matrix is variable but contains a combination of bacterial cells, proteins, enzymes, polysaccharides, lipids and nucleic acids [4,5]. Biofilms provide a protective structure for bacteria, allowing them to grow in hostile environments [1,6]. Since they can form on both living and nonliving surfaces they can cause serious problems for different industries, leading to reduced product quality and economic losses [7,8,9]. Bacteria growing in biofilms are more resistant to stress factors, such as temperature, pH and disinfectants, than are their planktonic counterparts [10]. Generally, biofilms grow in damp places where they have access to nutrients [11,12]. In the dairy industry, the main source of nutrients for bacteria is left-over product on equipment; the biofilms that grow then cause the microbial contamination of the next batch of product [13,14].

Foodborne diseases remain a major threat to public health and the economy. Indeed, food intoxication is among the most important causes of mortality and morbidity [15], with approximately 42,000 deaths and 600 million cases of illness each year [8,16]. The great majority of problems are of bacterial origin, with contamination of food during manufacturing and preconsumption storage, the usual routes of bacterial entry. In the food industry, most, but not all, biofilms are formed by nonpathogenic bacteria [17]. The resistance of biofilms to chemical/physical cleaning and sanitizing procedures renders those that contain pathogens a definite health hazard [8,11]; certainly, biofilms that act as reservoirs of biogenic amine (BA)-producing bacteria pose a food safety problem [18].

BAs are compounds of low molecular weight, organic and nitrogen-containing that are synthesized by the enzymatic decarboxylation of specific amino acids [19,20]. In many organisms, they have important biological functions [21,22]. However, in foodstuffs they can accumulate in large quantities (especially fermented foods and beverages) owing to the metabolism of certain microorganisms, and this can cause health problems [23,24]. In fermented dairy products, BAs are mainly formed by certain lactic acid bacteria (LAB) present in the starter culture or introduced as contaminants during manufacturing [25].

One of the most dangerous BAs in dairy products is histamine; the ingestion of cheese with high concentrations of histamine can lead to toxic neurological, gastrointestinal and respiratory effects [26,27,28]. Indeed, after fish, the food most commonly at the root of histamine poisoning is cheese. Problems have been recorded after the consumption of different types of cheese, including Swiss and Cheddar [29].

*Lentilactobacillus parabuchneri* has been identified as the species largely responsible for histamine production and accumulation in dairy products [30]. It is part of the non-starter culture microbiota and has been found in numerous cheese varieties, including Caciocavallo Pugliese, Spanish farmhouse cheese, Parmigiano Reggiano, Camembert, Gouda-type cheese, Pecorino Crotonese, Cheddar, Emmental and Swiss goat milk cheese [30,31]. Some *L. parabuchneri* strains influence the organoleptic characteristics of cheese, such as eye formation, during the later stages of ripening [32]. However, most of the strains studied so far have been found to produce histamine as well [33,34,35]. Moreover, *L. parabuchneri* has consistently been shown present on different dairy machinery surfaces, resulting in contaminated milk and cheese, the latter eventually containing high concentrations of histamine [31,36]. Diaz et al. [37] reported some histamine-producing strains of *L. parabuchneri* to form biofilms, which explains the apparent ease which they persist in dairy facilities. However, little is known about the nature of these biofilms or of the conditions that favor their formation.

The aim of the present work was to investigate biofilm formation by different *L. parabuchneri* strains, to determine the composition of the biofilm matrices produced, and to examine the influence on biofilm formation of temperature, pH, NaCl concentration, carbon source and surface type.

## 2. Materials and Methods

### 2.1. Bacterial Strains and Culture Conditions

Table 1 lists the 25 *L. parabuchneri* strains used in this study—the type strain plus 24 strains of dairy origin. All were cultured in MRS broth (Oxoid, Basingstoke, Hampshire, UK). Unless otherwise indicated, incubation proceeded at 37 °C under anaerobic conditions (10% H_2_, 10% CO_2_ and 80% N_2_) in a Mac 1000 anaerobic workstation (Don Whitley Scientific, Shipley, UK) with the temperature and gas concentration under automatic control [38]. To analyze the influence of the carbon source on biofilm formation, however, the medium employed was MRS broth without dextrose (US Biological, Salem, MA, USA), supplemented with glucose, galactose or lactose as required (2% *w*/*v*). The strains were pre-grown in this medium before analyzing their biofilm forming capacity. To study the effect of NaCl concentration on biofilm formation, MRS broth was supplemented with 1%, 2%, or 3% *w*/*v* NaCl.

### 2.2. Biofilm Formation Capacity of L. parabuchneri Strains

Although the capability to form biofilm was previously studied in some of the strains examined in this work, (indicated with a reference in Table 1), to compare results and categorize them, they were included in the present study. The *L. parabuchneri* strains were initially categorized in terms of their ability to form biofilms on polystyrene using the crystal violet method [41]. Briefly, overnight cultures in MRS broth were diluted to a concentration of 10^6^ CFU/mL. A total of 200 µL were then inoculated into the wells of a 96-well (round-bottomed) microtiter plate (Nunc MicroWell Plates with a Nunclon Delta Surface) (preparing at least 3 biological and 2 technological replicates for all experiments, with experiments performed in at least triplicate). As a negative control, sterile medium was used.

These plates were routinely incubated at 37 °C for 48 h. However, to analyze the influence of time and temperature on biofilm formation, they were incubated for 12, 24, 48, 72 and 96 h, and at 8, 12 and 24 °C for 2, 7, 14 and 30 days, respectively. To examine the influence of an acidic environment, the strains were incubated in MRS broth at pH 4.7 (and at pH 6 as a control) under standard time and temperature conditions. The biomass of the biofilm detected using the crystal violet technique was quantified as described by Diaz et al. [18], with slight modifications. After incubation the supernatant was removed and the wells washed twice with 200 µL of PBS buffer to eliminate nonadherent cells. All the wells were then air-dried for 30 min at room temperature in a CRUMAir 9005-FL laminar flow cabinet (CRUMA, Barcelona, Spain). The biofilms formed were stained with 250 µL of 0.5% (*w*/*v*) crystal violet diluted with distilled, sterilized water (dH_2_O) for 30 min at room temperature. The nonbound dye was eliminated and the wells washed 3 times with 200 µL of dH_2_O. Finally, the bound dye was solubilized with 250 µL of acetic acid (33% *v*/*v*) and the absorbance measured at 595 nm using a Benchmark Plus microplate spectrophotometer (BioRad, Hercules, CA, USA). The biomass of the biofilm formed under each set of conditions was expressed as the mean of the results for the biological and technical replicates. The ability to produce biofilm was expressed using cut-off values [42,43]. The mean of the optical density (OD) ± SD for the 3 replicates was calculated for each strain. The mean for the negative controls, noninoculated media from the same batch, including biological and technical replicates (ODnc) was used to determine the cut-off values used to define the different categories of biofilm producers (ODc). These threshold values were calculated as the ODnc value plus 3 SDs. This allowed the strains to be classified into different categories:

- ODc < OD ≤ 2 × ODc = weak producer
- 2 × ODc < OD ≤ 4 × ODc = moderate producer
- OD > 4 × ODc = strong producer.

The OD value is the average value of biological and technical replicates for each strain.

### 2.3. Biochemical Composition of Biofilm Matrices: Dispersal Assays

Bacterial biofilms were prepared in 96-well microtiter plates as described above. After 48 h of incubation, nonbound cells were removed and the wells washed once with PBS buffer. The adhered biofilm matrix was then treated as previously described [44,45], adding 200 µL per well of different enzyme suspensions: DNAseI (100 µg/mL in 150 mM NaCl; 1 mM CaCl_2_) to degrade extracellular DNA (eDNA); proteinase K (100 µg/mL) and trypsin (100 µg/mL [in 20 mM Tris-HCl pH 7.5; 100 mM NaCl]) to degrade proteins; RNAse (10 mg/mL [in 5 mM of MgCl_2_]) to degrade RNA; and 10 mM sodium periodate (NaIO_4_ [in 50 mM sodium acetate buffer pH 4.1]) to degrade exopolysaccharides (EPSs). Any biofilm dispersal (with the accompanying loss of biomass) indicated the presence of the corresponding substrate in the matrix. Control wells were filled with PBS buffer without enzymes.

All plates were incubated at 37 °C for 24 h. The enzyme mixture was then removed and the biofilms washed once with PBS, dried and stained with 0.5% crystal violet as described above. Biofilm dispersal was assessed by measuring the absorbance at 595 nm. Three biological replicates were made for each sample, and each experiment repeated at least 3 times.

### 2.4. Bacterial Adherence to Different Surfaces

Bacterial adherence to different surfaces was examined using 1 cm^2^ ‘coupons’ of each material. These included food-grade stainless steel (type AISI 304), beech wood, rubber and food-grade plastic, i.e., the most typical surfaces found in the dairy industry [10,46,47,48]. All were cleaned and sterilized in an autoclave prior to use.

100 µL of each strain suspension (10^9^ CFU/mL) were then added to a tube containing a sterile coupon in 10 mL of MRS. For each material, a tube without cells was used as negative control. All tubes were incubated at 37 °C for 48 h. Any nonadhered cells were then removed by rinsing the coupon twice with 2 mL of PBS buffer. The biofilm produced on 1 of the faces of the coupon was then removed with a sterilized swab and immersed in 1 mL of PBS. The number of adhered bacteria was determined by plating serial dilutions on MRS [18]. All experiments were performed with 3 replicates for each strain and material, employing independent cultures. The results were expressed as log_10_ CFU (mean ± SD of the replicates).

### 2.5. Scanning Electron Microscopy Images

Biofilm formation on different surfaces was observed as previously described [41] with some modifications. Briefly, the strains were incubated for 48 h at 37 °C in tubes with 10 mL of MRS medium containing the above-described coupons (1 × 1 cm^2^). After incubation, the coupons were cleaned twice with PBS buffer and fixed in 2.5% glutaraldehyde (Sigma–Aldrich, Munich, Germany) in PBS for 16 h at room temperature. The fixed biofilms were then dehydrated using a graded series of acetone solutions (50–100% *v*/*v*), dried with argon, coated with platinum (using a SCD 005 sputter coater) and observed using a dual-beam FIB/SEM system (Quanta 3D FEG, FEI Company, Eindhoven, NL, USA).

### 2.6. Data Analysis

Biofilm formation at different times, temperatures and on different surfaces was compared by 2-way analysis of variance (ANOVA) followed by a Bonferroni post-hoc test. The Student *t* test were used to compare the effect of pH, NaCl concentration and carbon source, on biofilm formation. The same test was used to compare the effect of the different enzyme treatments on the biofilm matrix. All calculations were performed using SPSS Statistics v.15.0 software. Significance, unless otherwise indicated, was set at *p* < 0.05.

## 3. Results

### 3.1. Biofilm Formation by L. parabuchneri Strains

**Figure 1:**
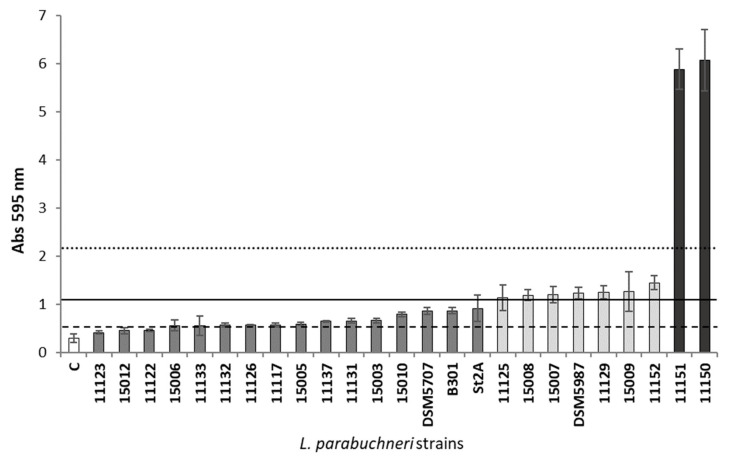
Biofilm-producing capacity on polystyrene of the histamine-producing *Lentilactobacillus parabuchneri* strains. The strains were incubated at 37 °C for 48 h. Data represent means ± SD (error bars) of at least three independent experiments. Shading of the same color indicates no significant difference. The producer/nonproducer cut-off (weak producer) is signaled by the dashed line (ODc), the solid line is 2 × ODc (moderate producer) and the dotted line 4 × ODc (strong producer).

The crystal violet technique categorized the strains as either weak, moderate or strong biofilm producers (Figure 1). Two strains belonging to each category, *L. parabuchneri* IPLA11117 and 11122 (weak producers), 11125 and 11129 (moderate producers) and 11150 and 11151 (strong producers), were selected for further work.

### 3.2. Biochemical Composition of Biofilms

**Figure 2:**
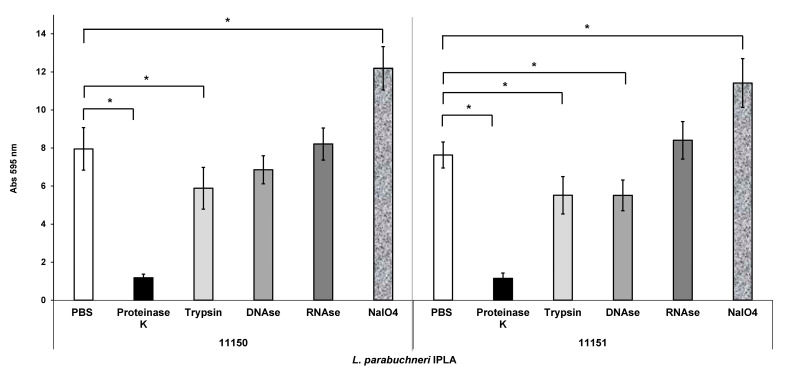
Effect of proteinase K, trypsin, DNAse I, RNAse and NaIO_4_ on biofilms produced by the strong biofilm producers *L. parabuchneri* IPLA11150 and IPLA11151. The strains were incubated for 48 h at 37 °C and subjected to treatments with the stated agents for 24 h. Data represent means ± SD (error bars) of three experiments. Values marked with * differ significantly.

*L. parabuchneri* IPLA11150 and IPLA11151, classified as strong biofilm producers, were chosen to produce biofilms for matrix analyses. Assaying biofilm dispersal is the main method used to infer the components involved in biofilm matrix. Mature biofilms were treated with proteinase K, trypsin, DNAse I, RNAse or NaIO_4_ (and PBS buffer as a negative control) as previously described [45,49,50]. After 48 h, the biofilms formed in microwell plates were subjected to each of the aforementioned treatments for 24 h (Figure 2). For both the IPLA11150 and IPLA11151 strains, the proteinase K treatment had the greatest effect in terms of the dispersal of the biofilm, most probably due to its broader spectrum of cleavage. Exposure to trypsin (another protease) also led to a significant dispersal, although less strong. For IPLA11151, DNAse I only slightly dispersed the biofilms. No significant effect was observed for the RNAse treatment. In contrast, the NaIO_4_ treatment led to a significant increase in biofilm biomass formation for both strains.

### 3.3. Biofilm Formation after Different Incubation Times

**Figure 3:**
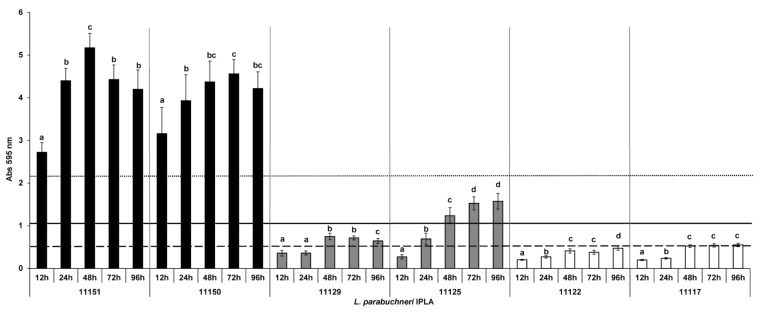
Biofilm-producing capacity on polystyrene at different times. The strains were incubated at 37 °C for 12, 24, 48, 72 and 96 h. Data represent means ± SD (error bars) of three experiments. For each strain, values marked with the same letter are not significantly different (*p* > 0.05 according to the Bonferroni post-hoc test). The producer/nonproducer cut-off (weak producer) is signaled by the dashed line (ODc), the solid line is 2 × ODc (moderate producer) and the dotted line 4 × ODc (strong producer).

Biofilm formation by the selected strains was analyzed at different incubation times. For most of the assayed strains, biofilm biomass was at a maximum after 48 h of incubation (Figure 3). The exceptions were the moderate biofilm-producing strain IPLA11125 and the weak biofilm producer IPLA11122, which reached a maximum biofilm biomass after 72 h and 96 h of incubation, respectively. However, after 48 h, both had reached biomass values within the range of their established biofilm-producer categories, and the increment observed after 72 or 96 h did not reach the threshold required to change category. Consequently, the incubation time was fixed at 48 h for all assays. It is noteworthy that *L. parabuchneri* IPLA11151 and 11150 produced biofilms after just 12 h of incubation.

### 3.4. Biofilm Formation at Colder Temperatures

**Figure 4:**
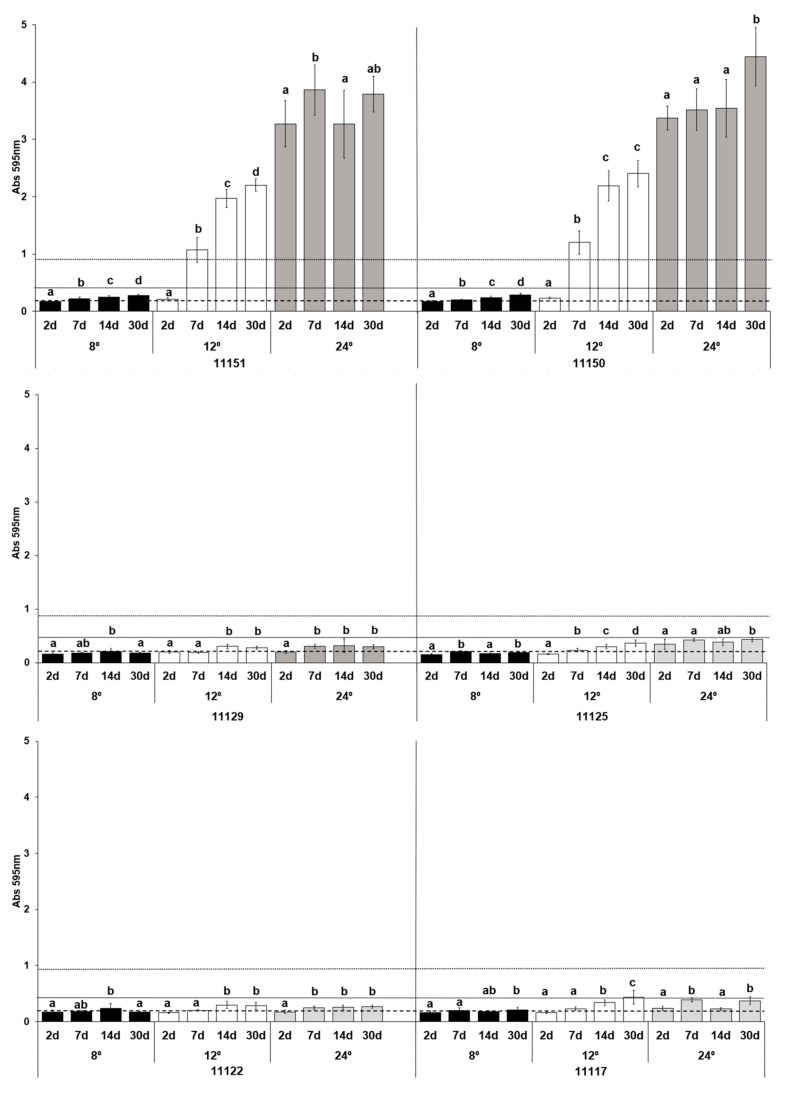
Biofilm-producing capacity on polystyrene of the histamine-producing *L. parabuchneri* strains incubated at 8, 12 and 24 °C. The strains were incubated for 2, 7, 14 and 30 days. Data represent means ± SD (error bars) of three experiments. Values marked with the same letter do not differ significantly (*p* > 0.05 according to the Bonferroni post-hoc test). The producer/nonproducer cut-off (weak producer) is signaled by the dashed line (ODc), the solid line is 2 × ODc (moderate producer) and the dotted line 4 × ODc (strong producer).

The influence of cold temperatures on biofilm formation was examined given the importance of refrigeration for dairy products. Since refrigeration reduces microbial growth, the incubation times were extended (Figure 4). In general, all the analyzed strains showed a reduction in their ability to form biofilms at 8 °C. None of them, not even the strong producers IPLA11150 and IPLA11151, produced biofilm at this temperature, even after 30 days of incubation. Indeed, significant differences (*p* < 0.001) were seen between mean biofilm biomass formation after 30 days at 8°, 12° and 24 °C for *L. parabuchneri* IPLA11151 (0.28 ± 0.19; 2.20 ± 0.11; 3.79 ± 0.31, respectively), IPLA11150 (0.29 ± 0.02; 2.40 ± 0.23; 4.44 ± 0.51) and IPLA11125 (0.19 ± 0.01; 0.37 ± 0.06; 0.43 ± 0.04). For the other three strains, biofilm formation after 30 days of incubation at 8 °C differed significantly with that seen at 12 °C and 24 °C (*p* < 0.001): for *L. parabuchneri* IPLA11129 0.18 ± 0.01 compared to 0.28 ± 0.02 and 0.29 ± 0.03, respectively; for IPLA11122 (a weak biofilm producer) 0.18 ± 0.01 compared to 0.28 ± 0.06 and 0.27 ± 0.03, respectively; and for IPLA11117 0.21 ± 0.05 compared to 0.44 ± 0.12 and 0.38 ± 0.07, respectively. In general, biofilm formation was clearly greater at 24 °C than at lower temperatures.

Interestingly, strong biofilm-producing strains reached their category threshold value even at 12 °C. At this temperature they needed seven days to reach maximum production, while they did so after only two days at 24 °C. The moderate and weak biofilm-producing strains showed a reduction in biofilm-forming capacity at the intermediate temperatures assayed. *L. parabuchneri* IPLA11125 and IPLA11129 were not even able to reach the minimum absorbance value for the category to which they belonged (moderate).

Although, the ability of *L. parabuchneri* to produce biofilms at colder temperatures was strain dependent, the strong biofilm producers formed notable biofilm biomass at 12 °C and 24 °C, a result of some importance given the temperatures associated with cheesemaking, especially during ripening [51,52,53].

### 3.5. Biofilm Formation in an Acidic Environment

**Figure 5:**
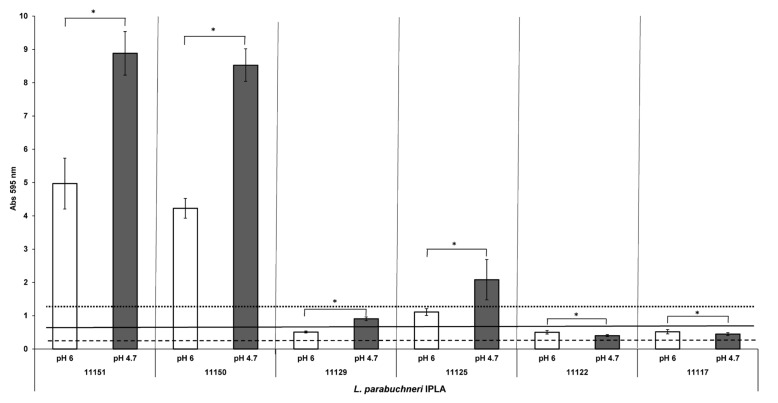
Biofilm-producing capacity on polystyrene of the biogenic amine-producing *Lentilactobacillus* strains incubated with MRS at pH 4.7 and 6. The strains were incubated for 48 h at 37 °C. Data represent means ± SD (error bars) of three experiments. Values marked with * differ significantly. The producer/nonproducer cut-off (weak producer) is signaled by the dashed line (ODc), the solid line is 2 × ODc (moderate producer) and the dotted line 4 × ODc (strong producer).

Milk acidification is an important phenomenon during the manufacture of fermented dairy products. The effect of an acidic pH (4.7) on biofilm formation in MRS broth was therefore analyzed for all six selected strains, comparing the results to those obtained at pH 6 (the normal pH of MRS). The biofilm biomass increased very significantly during cultivation in the acidic broth (Figure 5). The largest increment—almost double the biofilm biomass measured as OD—was recorded for the strong biofilm producers. However, *L. parabuchneri* IPLA11125, a moderate producer, also behaved like a strong biofilm producer at the acidic pH (Figure 5). A significant increase in biofilm biomass was also observed for the other moderate producer IPLA11129, although not as for IPLA11125. In contrast, the weak biofilm-producing strains showed a reduced capacity to form biofilms at pH 4.7.

### 3.6. Influence of Carbon Source on Biofilm Formation

**Figure 6:**
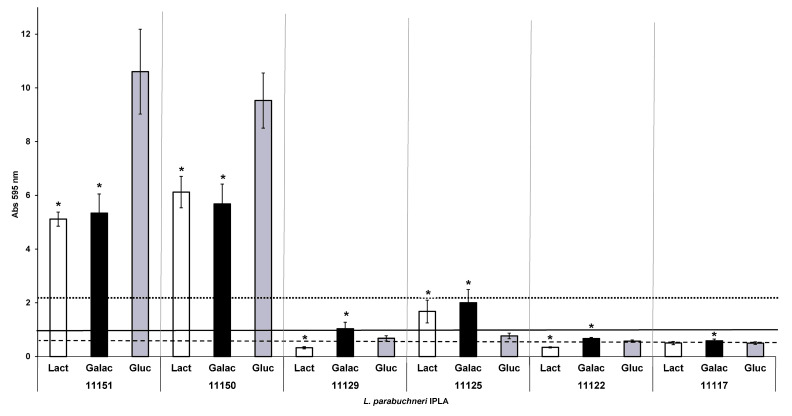
Biofilm-producing capacity on polystyrene of the histamine-producing *L. parabuchneri* strains incubated with MRS supplemented with different carbon sources. The strains were incubated for 48 h at 37 °C. Data represent means ± SD (error bars) of three experiments. Values marked with * differ significantly, considering glucose as the control. The producer/nonproducer cut-off (weak producer) is signaled by the dashed line (ODc), the solid line is 2 × ODc (moderate producer) and the dotted line 4 × ODc (strong producer). Lact: Lactose; Galac: Galactose; Gluc: Glucose.

The most abundant sugar in the dairy environment is lactose, which is metabolized to glucose and galactose by β-galactosidases. The influence of these three sugar sources on biofilm formation by the six selected *L. parabuchneri* strains was studied (Figure 6). For the strong producers, biofilm formation was greatest in the presence of glucose (standard condition). With lactose and galactose, their production of biofilm biomass was less strong, although they still returned biomass values exceeding the threshold value for strong biofilm producers. Both moderate producers showed an increase in biofilm formation in the presence of galactose. However, when lactose was the carbon source, IPLA11125 showed an increase in biofilm biomass formation, while for IPLA11129, production was close to that of a weak biofilm producer. For the weak producer IPLA11122, biofilm formation increased with lactose and galactose in comparison with glucose, while for IPLA1117, it increased only with galactose. However, in both cases, the production values were still those of weak producers, or indeed, under the threshold for recognition as a biofilm producer.

### 3.7. Effect of NaCl on Biofilm Formation

**Figure 7:**
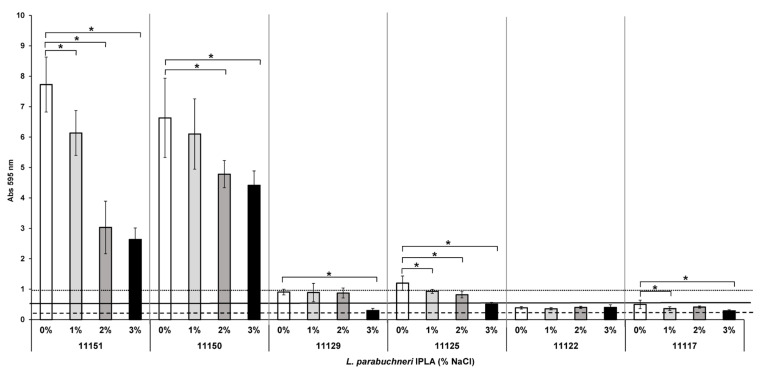
Effect of NaCl concentration (in % *p*/*v*) on biofilm-producing capacity on polystyrene of the histamine-producing *L. parabuchneri* strains. The strains were incubated for 48 h at 37 °C. Data represent means ± SD (error bars) of three experiments. Values marked with * differ significantly. The producer/nonproducer cut-off (weak producer) is signaled by the dashed line (ODc), the solid line is 2 × ODc (moderate producer) and the dotted line 4 × ODc (strong producer).

During cheesemaking, NaCl is added in variable quantities at different times, either directly or by immersion in brine. The effect of different NaCl concentrations on the biofilm formation capacity of the six *L. parabuchneri* strains was therefore analyzed (Figure 7). This capacity was reduced in all the analyzed strains as the NaCl concentration increased. For the strong producers, the inhibitory effect was greater on IPLA11151 than on IPLA11150 (not seen until a salt concentration of 2% was tested). The moderate producers were also inhibited, more so *L. parabuchneri* IPLA11125 than IPLA11129 (no reduction recorded until 3% NaCl was tested). At the concentrations tested, NaCl had a small effect on the weak producer IPLA11117 and no significant effect on the other weak producer IPLA11122.

### 3.8. Bacterial Adherence to Stainless Steel, Food-Grade Plastic, Beech Wood and Rubber

All six tested strains adhered to all the surfaces tested (adhesion capacity was expressed via the number of viable cells adhered to the surfaces [CFU/mL cm^2^]). Apart from the control polystyrene, beech wood was the material to which all the strains adhered best. The strong producer *L. parabuchneri* IPLA11151 adhered similarly well to all the surfaces studied, suggesting it poses a risk of forming biofilms throughout the cheese production process. The other strong biofilm producer, IPLA11150, adhered similarly well to beech wood and polystyrene, but log-reduced values were recorded for the other surfaces. The moderate producer IPLA11129 showed similar adhesion values for polystyrene, beech wood and plastic and slightly reduced values for stainless steel and rubber. Surprisingly, IPLA11125 (a moderate producer), IPLA11122 and IPLA11117 (weak producers) returned strong adhesion values for all surfaces, but especially for polystyrene and beech wood, with values similar to those recorded for the strong producers (Table 2). Thus, biofilm formation depends on more than cell adhesion capacity alone.

Overall, no significant differences were seen between the tested strains with respect to adhesion to polystyrene and beech wood, confirming that these materials facilitate cell attachment. However, with respect to stainless steel, cell adhesion was higher for IPLA11151, IPLA11125 and IPLA11122. The value recorded for IPLA11117, in contrast, was half that recorded for IPLA11151. The strong producer IPLA11151 and the moderate produced IPLA11129 showed the highest adherence values with respect to plastic. Finally, for rubber, the highest adhesion values were returned by the strong producer IPLA11151 and the moderate producer IPLA11125, again showing that factors other than cell attachment are involved in biofilm formation.

### 3.9. SEM Analysis of Biofilm Formation on Different Surfaces

**Figure 8:**
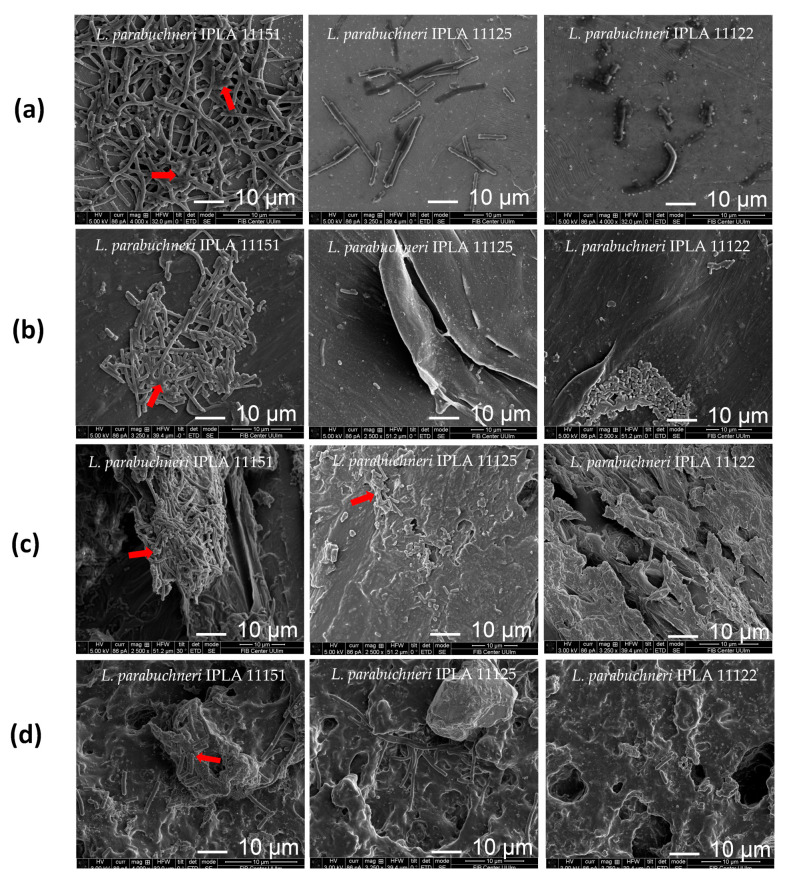
Scanning electron microscopy images of *L. parabuchneri* strain biofilms grown at 37 °C for 48 h on different surfaces: (**a**) stainless steel; (**b**) food-grade plastic; (**c**) beech wood; and (**d**) rubber. Scale bars (10 μm) are shown on the photomicrographs. Red arrows indicate extracellular polymeric substances in the matrix.

Scanning electron microscopy (SEM) photomicrographs were made of the biofilms formed by the strong producer IPLA11151, the moderate producer IPLA11125 and the weak producer IPLA11122. Images of the coupons were taken after 48 h of incubation in MRS medium (Figure 8). The images show the visual characteristics of each *L. parabuchneri* strain and its ability to attach to each of the surfaces, which have different roughness and permeability properties, etc. The stainless steel and plastic surfaces were clearly smoother than the beech wood and rubber surfaces (adhesion was more easily achieved on these rougher and more hydrophobic surfaces). SEM is frequently used to observe biofilm spatial structure and to detect the presence of extracellular polymeric substances [54]. In the present work, some cells produced such polymers around themselves after 48 h of incubation, and some cells were elongated. This indicates that the biofilms had progressed beyond the first stages of attachment.

Interestingly, EPSs provide mechanical stability to biofilms and help in the development of three-dimensional spatial structures that influence functional properties, such as resistance to antimicrobial agents and cleaning treatments [4]. In the present work, EPS production was greater in the strong producer IPLA11151 than in either of the other two strains tested. This was especially true with respect to the stainless steel, plastic and rubber coupons (Figure 8).

## 4. Discussion

In the food industry, microbial biofilms are of great concern given their connection with food safety and quality [2,12,13,54]. The great majority of foodborne diseases are of bacterial origin, with the contamination of food during manufacturing and preconsumption storage the usual routes of bacterial entry and development. *L. parabuchneri* is known to be largely responsible for the production and accumulation of histamine in cheese [30]. Histamine is one of the most toxic of BAs [23,28] and the only one for which a legal limit has been established, although only for certain foods [23,55]. In fact, there is no specific regulation regarding its concentration in dairy products, not even for cheese, in which it can reach high concentrations [56]. Reducing the presence of *L. parabuchneri* in cheese is, however, recommended [25,37]. Some histamine-producing strains of *L. parabuchneri* are also known to produce biofilms in the dairy environment [18,37], which could act as reservoirs of food-contaminating microorganisms that subsequently cause the accumulation of histamine at undesirable concentrations [18,31]. This phenomenon is especially problematic with respect to cheeses that are processed post-ripening for market presentation (cut, sliced or grated). Biofilms can form on the surface of the equipment used [57], from which histamine-producing bacteria can contaminate the cheese being processed [58].

Significant strain-specific differences in biofilm formation have been observed for well-known biofilm-forming species such as *S. aureus* and *Salmonella* spp. [59]. The present work examined and compared the biofilm-forming capacity of 24 histamine-producing *L. parabuchneri* strains of dairy origin (plus the type of strain included as a reference). All were able to form biofilms and were classified as either strong, moderate or weak producers based on their ability to produce a biofilm on polystyrene. More than one-third of the analyzed strains were classified as moderate or strong producers (Figure 1). This would give these strains an advantage in colonizing surfaces at dairy facilities and represents a threat to food safety. Two strains from each category were selected for further study: *L. parabuchneri* IPLA11150 and 11151 as strong producers, IPLA11125 and 11129 as moderate producers and IPLA11117 and 11122 as weak producers.

Biofilm dispersal assays are the method most commonly used to determine the components involved in biofilm matrix. A reduction in biofilm biomass was seen for the strong biofilm producers, IPLA11151 and 11150, after treatment with proteinase K and trypsin. These proteases recognize different protein substrates, suggesting that the biofilm matrix is composed mainly of proteins (Figure 2). In addition, the biomass of the biofilm produced by IPLA11151 was reduced by treatment with DNAse I, suggesting that eDNA is also present in its biofilm matrix. In some food spoilage *Lactobacillus plantarum* strains, the biofilm matrix was also shown to be mainly formed by protein and eDNA, although differences in the importance of both compounds between strains were also observed [60]. However, no effect was observed for either IPLA11150 or IPLA11151 after treatment with RNAse, suggesting RNA to be absent from their biofilms. NaIO_4_ was used as a dispersal agent to test if EPSs were present, but its use actually led to an increase in biomass. This might be due to the effect of NaIO_4_ on EPSs that are chemically identical in structure, but that have differences in terms of the acetates O-linked to succinate, or the acetylation levels of amino groups [61,62]. In biofilms, polysaccharides may be segregated or associated with other molecular species such as DNA, proteins and lipids, with which they can interact [63]. As a consequence, the depolymerization of EPSs in response to NaIO_4_ varies depending on a biofilm’s composition. Dakheel et al. [44] reported that NaIO_4_ could show strong to weak biofilm dispersal capacity, revealing different patterns of interaction between EPSs and proteins. Sager et al. [64] showed that NaIO_4_ had a stimulating influence on established biofilms of *Pasteurella pneumotropica*, an effect similar to that seen in the present work, indicating the presence of EPSs associated with other components that affect its biofilm dispersing capacity. However, with the techniques used in the present work, it is difficult to know what the precise involvement of EPSs in the biofilm matrix of *L. parabuchneri* may be.

The biofilm producing capacity of microbes is influenced by factors such as the attachment surface, the temperature, the presence of other species, and nutrient availability, etc. [13,65,66]. New materials that might prevent microbial growth are being explored [67,68], including the use of safe natural antimicrobial coating surfaces [69], but there remain many that are difficult to clean. The design requirements of equipment can also render effective cleaning and disinfection difficult [36]. In the present work, the adherence of the tested strains to materials commonly used in the dairy industry (and traditional facilities)—stainless steel, beech wood, food-grade plastic and rubber—was investigated. In other biofilm-forming species, such as *L. monocytogenes*, the surface material was found to be a major factor affecting biofilm production and with variations seen between strains [70]. The present histamine-producing strains of *L. parabuchneri* were all able to form biofilms on all the surfaces tested. The categorization of the strains as strong, moderate or weak produces (as determined on polystyrene using the crystal violet technique) was not upheld on the plastic and rubber surfaces, for which no significant differences in the numbers of adhered cells were seen (Table 2).

SEM images showed the beech wood to be the material on which the biofilms reached the greatest biomass (Table 2; Figure 8). On this material, even the weak biofilm producers were able to adhere in numbers similar to those recorded for the two strains classified as strong producers. This might be related to the roughness of this material, which was easily observable in the images (Figure 8). Although beech wood is not often used in large industrial facilities, it is still used for shelving in some traditional settings, e.g., where ripening occurs in environments such as natural caves. Rubber is a critical material in the industry, usually found at the connections between pipes and storage tanks; the capacity of histamine-producing *L. parabuchneri* strains to adhere to this material is a problem given the difficulties in its cleaning. In fact, this was one of the main localizations in which *L. parabuchneri* was located in some dairy facilities [36]. Stainless steel would seem to be the most appropriate material to use in the dairy industry since adhesion to this surface was apparently more difficult for all the analyzed strains, with the exception of the strong producer IPLA11151. It is also easier to clean and disinfect stainless steel than any of the other materials assayed, and it can better resist more extreme or abrasive treatments for biofilm elimination. Anyhow, the fact that some strains, such as *L. parabuchneri* IPLA11151 shown a greater adhesion capability indicates the need to maintain and maximize cleaning procedures to avoid the risk of contamination.

The SEM images revealed differences in roughness and porosity of the examined surfaces. Beech wood and rubber were the most porous materials explaining the enhanced cell adhesion values (i.e., especially beech wood). After 48 h of incubation, cells of all the three analyzed strains (IPLA11151, 11125 and 11122 [strong, moderate and weak producer, respectively]) were adhering to all the surfaces. During biofilm formation, cells elongate to connect adjacent microcolonies and to produce EPSs [44,71]. In the present work, the strong producer IPLA11151 yielded longer cells and larger amounts of EPSs. Indeed, the EPSs in biofilms plays a critical role in providing mechanical stability and in the formation of 3D spatial structures [72]. These structures aid in providing functional properties such as the ability to resist antimicrobial agents and cleaning treatments [43].

One of the most important factors affecting bacterial attachment to a given surface is the temperature of the environment. High temperatures make the surface of bacteria more hydrophobic, facilitating their tight binding to surfaces [10]. In the present work, cold temperatures reduced the capacity to form biofilms for all the tested strains (Figure 4). At 8 °C (refrigerator temperature), biofilm production over 30 days was most reduced for the ostensibly strong producers. The reduction effect on biofilm formation at reduced temperatures seems to be a general effect, as observed in other foodborne pathogens or spoilage bacteria [60,73,74], that could be related to a lower growth rate. Keeping production and storage temperature as low as possible could therefore improve food safety. In the manufacture of cheese this is not always possible [28,31], but it could still be important during post-ripening processing and storage. In the case of medium and weak biofilm producer strains, the reduction in incubation temperature has a prevention effect, making longer incubation times necessary, up to 30 days, to get closer to the absorbance values obtained at optimal temperature. However, in the case of strong biofilm producer strains, they reach the threshold value at 48 h at 24 °C and in seven days at 12 °C. It should be remembered that *L. parabuchneri* can still produce histamine at low temperatures [75], but in a temperature-dependent manner. Thus, by reducing the risk of biofilm formation and of histamine accumulation, maintaining a low temperature would have a doubly positive effect in terms of food safety.

pH also influences the ability of *L. parabuchneri* to produce biofilms. When incubated at pH 4.7, the moderate and strong producers returned greater biofilm biomass values than when incubated at pH 6 (Figure 5). Unfortunately, in the dairy environment, where the pH is usually low due to the production of lactate during lactose fermentation, the ability of *L. parabuchneri* to form biofilms would be favored. In *E. coli* acidic pH (5.5) also enhances the ability to form biofilm, although this effect was temperature dependent, at optimal growth temperature the acidic pH enhances the biofilm formation, while at restrictive temperature the biofilm formation in acidic conditions was reduced [76]. Probably this reduction effect was linked to a lower growth rate in nonoptimal environmental conditions. Biofilms offer resistance to acid stress, and some LAB are reported to survive acidic environments because of their ability to form biofilms [42,77,78].

In the dairy environment, the most abundant carbon source is lactose, the main sugar in milk. However, as soon as manufacturing begins, lactose is catabolized to glucose and galactose by the action of β-glucosidases. In the present work, (Figure 6) the strong producers made more biofilm in the presence of glucose, while for the moderate and weak producers, galactose favored greater production. In a dairy isolate of *Staphylococcus epidermidis*, glucose also favors biofilm formation, enhancing their three-dimensional structure in comparison with lactose. This effect was associated with an increase in EPSs formation, although it was not similar for all the tested strains. Thus, biofilm production with respect to carbon source would seem to be strain dependent. This might be related to the ability to consume the different sugar moieties or a hierarchical preference for consumption, as shown for other LAB species [79]. Carbon source could also influence the formation of EPSs. In some dairy isolated lactobacilli, glucose increased the formation of EPSs [80], which could explain the higher biomass observed, but in the *L. parabuchneri* strains studied in this work, we could not assess the presence and influence of EPSs in the biofilm matrix (Figure 2).

Although increased salinity has been shown to increase EPSs and biofilm formation in some bacterial species [81], in *L. parabuchneri* it had an antimicrobial effect (Figure 7). A clear reduction in biofilm biomass was observed for all the tested strains as the NaCl concentration of the medium increased (Figure 7). Thus, NaCl could be used to reduce the presence of biofilms. However, there is a consumer demand for foods with less salt, given the latter’s potentially harmful effects on health [82,83]. Unfortunately, this has led producers to reduce the presence of NaCl during cheesemaking, and this has been shown to increase the risk of histamine accumulation [84].

## 5. Conclusions

As far as we know, this is the first report of the influence of technological factors on biofilm formation by histamine-producing *L. parabuchneri* strains isolated from cheese. The attachment of *L. parabuchneri* cells to surfaces varies between strains, and some of them were able to form strong biofilms, mainly composed of proteins and eDNA. An acidic environment, concomitant to fermented dairy products, was found to promote biofilm production by the moderate and strong biofilm-producing strains. Biofilm formation was shown to be reduced as incubation temperature is reduced. However, the strong biofilm producers, showed resistance to acidic pHs and capacity to form biofilm at cold temperatures (12 °C), together with their ability to form biofilm in stainless steel surfaces and in rubber, mainly present in pipes and tube connections, constitute a safety risk threat. Refrigeration could be an important preventive measure to reduce the risk of biofilm formation, but it should be maintained over time. The addition of salt was also shown to reduce the ability to form biofilm. The best combination of environmental factors, low temperature and adequate salt concentration, needs to be maintained during cheese production to prevent biofilms formed by *L. parabuchneri*. This, plus using methods of biofilm elimination, may be the best strategy for reducing the presence of histamine in cheese and other foods.

## Figures and Tables

**Table 1:** *Lentilactobacillus parabuchneri* strains used in this study.

Strain	Origin	Reference
B301	Emmental	[39]
DSM 5987	Cheese	DSMZ-German Collection of Microorganisms
IPLA11117	Zamorano	[18]
IPLA11122	Emmental	[37]
IPLA11123	Emmental	Molecular Microbiology Group IPLA-CSIC
IPLA11125	Emmental	[37]
IPLA11126	Emmental	[37]
IPLA11129	Emmental	[37]
IPLA11131	Emmental	[37]
IPLA11132	Emmental	[37]
IPLA11133	Emmental	Molecular Microbiology Group IPLA-CSIC
IPLA11137	Emmental	Molecular Microbiology Group IPLA-CSIC
IPLA11150	Cabrales	[18]
IPLA11151	Cabrales	[18]
IPLA11152	Zamorano	[18]
IPLA15003	Mozzarella	Molecular Microbiology Group IPLA-CSIC
IPLA15005	Mozzarella	[18]
IPLA15006	Mozzarella	Molecular Microbiology Group IPLA-CSIC
IPLA15007	Mozzarella	Molecular Microbiology Group IPLA-CSIC
IPLA15008	Mozzarella	Molecular Microbiology Group IPLA-CSIC
IPLA15009	Mozzarella	[18]
IPLA15010	Mozzarella	Molecular Microbiology Group IPLA-CSIC
IPLA15012	Mozzarella	[18]
St2A	Zamorano	[40]
DSM 5707T	Human saliva (type strain)	DSMZ-German Collection of Microorganisms

IPLA-CSIC: Instituto de Productos Lácteos de Asturias—Consejo Superior de Investigaciones Científicas. DSMZ: German Collection of Microorganisms and Cell Cultures GmbH.

**Table 2:** Viable cells of *L. parabuchneri* strains adhered to surface material coupons after 48 h of incubation at 37 °C.

	*L. parabuchneri* IPLA					
Material/Strain	11151	11150	11129	11125	11122	11117
Polystyrene	6.79 ± 0.74	6.68 ± 1.12	6.45 ± 1.18	6.93 ± 0.74	7.08 ± 0.76	6.90 ± 0.87
Stainless Steel	6.22 ± 1.03 ^ac^	4.07 ± 0.99 ^bc^*	4.29 ± 0.83 ^bc^*	4.6 ± 0.32 ^abc^*	5.05 ± 1.15 ^ab^*	3.18 ± 1.29 ^bc^*
Plastic	5.37 ± 0.93 ^a^	4.10 ± 0.68 ^ab^*	5.17 ± 0.69 ^a^	4.85 ± 0.64 ^ab^*	4.97 ± 0.65 ^ab^*	3.52 ± 1.24 ^b^*
Beech Wood	6.11 ± 0.37	5.53 ± 0.93	6.40 ± 0.55	6.01 ± 0.48	6.13 ± 0.20	5.49 ± 1.39
Rubber	5.58 ± 0.71 ^a^	4.32 ± 1.45 ^ab^*	4.53 ± 0.43 ^ab^*	5.21 ± 0.43 ^a^*	4.46 ± 0.86 ^ab^*	3.40 ± 1.12 ^b^*

Data are expressed as CFU mL^−1^ cm^2^ and represent the mean ± SD of three experiments. Significant differences, as determined by ANOVA (*p* ˂ 0.05 according to Bonferroni post-hoc test), in adhesion between the strains for each material are labeled with different letters. * denotes significant differences within strains with respect to the polystyrene control.

## Data Availability

Data are contained within the article.
